# Perspectives of patients who inject drugs on a needle and syringe program at a large acute care hospital

**DOI:** 10.1371/journal.pone.0297584

**Published:** 2024-02-15

**Authors:** Hannah L. Brooks, Kelsey A. Speed, Kathryn Dong, Ginetta Salvalaggio, Bernadette (Bernie) Pauly, Marliss Taylor, Elaine Hyshka

**Affiliations:** 1 School of Public Health, College of Health Sciences, University of Alberta, Edmonton, Alberta, Canada; 2 Department of Emergency Medicine, Faculty of Medicine & Dentistry, College of Health Sciences, University of Alberta, Edmonton, Alberta, Canada; 3 Department of Family Medicine, Faculty of Medicine & Dentistry, College of Health Sciences, University of Alberta, Edmonton, Alberta, Canada; 4 Canadian Institute for Substance Use Research, University of Victoria, Victoria, British Columbia, Canada; 5 Streetworks, Boyle Street Community Services, Edmonton, Alberta, Canada; Massachusetts General Hospital and Harvard Medical School, UNITED STATES

## Abstract

**Background:**

People who inject drugs in North America often continue to inject while hospitalized, and are at increased risk of premature hospital discharge, unplanned readmission, and death. In-hospital access to sterile injection supplies may reduce some harms associated with ongoing injection drug use. However, access to needle and syringe programs in acute care settings is limited. We explored the implementation of a needle and syringe program integrated into a large urban tertiary hospital in Western Canada. The needle and syringe program was administered by an addiction medicine consult team that offers patients access to specialized clinical care and connection to community services.

**Methods:**

We utilized a focused ethnographic design and semi-structured interviews to elicit experiences and potential improvements from 25 hospitalized people who inject drugs who were offered supplies from the needle and syringe program.

**Results:**

Participants were motivated to accept supplies to prevent injection-related harms and access to supplies was facilitated by trust in consult team staff. However, fears of negative repercussions from non-consult team staff, including premature discharge or undesired changes to medication regimes, caused some participants to hesitate or refuse to accept supplies. Participants described modifications to hospital policies regarding inpatient drug use or access to an inpatient supervised consumption service as potential ways to mitigate patients’ fears.

**Conclusions:**

Acute care needle and syringe programs may aid hospital providers in reducing harms and improving hospital outcomes for people who inject drugs. However, modifications to hospital policies and settings may be necessary.

## Introduction

Approximately 15.6 million people inject drugs globally and are at elevated risk of bloodborne infection, chronic morbidity, and premature mortality [1, 2]. Robust global evidence identifies needle and syringe programs (NSPs) as a key intervention for reducing these negative outcomes [3, 4]. NSPs provide people who inject drugs (PWID) with access to sterile drug consumption supplies and other supports, and have been implemented in over 80 countries and within various contexts (e.g., fixed and mobile sites, pharmacies, dispensing machines, and prisons) [5]. Meta-analyses and systematic reviews indicate that PWID who regularly attend an NSP have lower rates of injection-related risk behaviours and HIV seroconversion [6, 7]. NSPs also promote communication and trust between PWID and health care providers and facilitate uptake into health services [8, 9]. These benefits appear most impactful when an NSP is easily accessible and located close to where injection drug use occurs [3, 9, 10].

Despite abundant evidence and widespread implementation in community and outpatient settings, NSPs and other harm reduction interventions are not routinely available in hospitals [11, 12]. This is problematic because PWID experience high rates of hospitalization [11, 13] and cohort and survey data suggest that 30%-50% of hospitalized PWID continue to consume drugs while admitted [14–18]. In-hospital drug use is driven by several factors. Most physicians receive inadequate training on managing substance use disorders in their primary specialty, and access to inpatient addiction medicine specialists remains limited [19]. This is further compounded by limited medication options for people experiencing problematic stimulant use. Moreover, PWID frequently report insufficient pain and withdrawal management [20, 21], which can lead to discomfort and distress and a need to self-manage symptoms through drug use. Even when specialized care is available, a subset of PWID may continue to inject. Qualitative studies with PWID suggest that ongoing drug use may result from anxiety, stress, boredom, and stigma experienced during acute care episodes [20, 21].

For people who continue to inject drugs while hospitalized, hospitals can become a high-risk environment where they encounter more difficulty accessing NSP than in community settings [21]. Formal and informal bans on injecting in hospital result in constrained access to sterile injection supplies, which increases the risk of syringe sharing or reuse, and infection [15, 20, 22, 23]. Such restrictions on drug use can exacerbate well-documented mutual mistrust and poor communication between PWID and hospital staff [19, 24] and increase the risk of patient-initiated hospital discharge, involuntary discharge, unplanned readmission, and death [15, 17, 25–27]. Similar to impacts seen in community settings, inpatient NSPs may improve patient outcomes by facilitating a reduction of injection-related infections, supporting patients to complete treatment, and engendering communication and trust between patients and staff [9, 10].

In the United States, empirical research describes an NSP located adjacent to a hospital [28, 29] and the distribution of supplies to hospitalized patients upon discharge [30]. In Canada, the distribution of supplies has been noted to occur in one acute care facility [31], within specialized sub-acute care facilities [32, 33], and sporadically within emergency departments [34]. However, data on the perspectives of patients who accepted NSP supplies are lacking. To address this gap, we undertook a mixed-method study to assess the implementation of an NSP integrated within an urban, 850-bed hospital in Western Canada.

### Overview of the NSP

The NSP is operated by a multidisciplinary addiction medicine consult team (AMCT). The AMCT offers all patients access to immediate and comprehensive pain management; pharmacotherapy for substance use disorders, including opioid agonist treatment (specifically buprenorphine/naloxone, methadone and slow-release oral morphine) and withdrawal management; inpatient addiction counselling and peer support; enhanced social care including connections to housing and income support; health promotion interventions (e.g., immunizations); and referrals to primary care and other community-based addiction treatment providers [35]. The NSP was implemented in 2014, after AMCT leadership reviewed hospital data demonstrating that inpatients were contracting recurrent infections due to ongoing drug use with non-sterile drug consumption supplies, and consulted with PWID who described a need for sterile supplies to be distributed at the hospital. AMCT patients who report recent intravenous drug use are offered needles, syringes, tourniquets, sterile water, alcohol swabs, acidifiers, and cookers to store within their belongings. Patients are then provided safer drug-use education, personal sharps containers, and storage and disposal instructions, and at the time this study was conducted, were warned that drug use was not permitted anywhere on hospital property. Members of the AMCT note the provision of supplies in the patient’s medical chart, which is accessible to all hospital staff.

Below we report qualitative findings from a larger mixed-method process evaluation of the NSP conducted by an academic research team affiliated with the AMCT (EH, HLB, KAS, GS are or were members of the research team, KD was medical director of the AMCT during the study). In the first phase of our evaluation, we abstracted quantitative data from patient and program records to estimate the frequency that patients were offered and accepted supplies and to identify the main demographics that predicted NSP uptake. Of 334 records where inpatients were offered NSP supplies, 37% (n = 124) accepted [36]. To understand barriers and facilitators to NSP uptake, the second phase of our evaluation included qualitative research with AMCT patients and hospital staff that provide care for PWID. Herein, we report findings from qualitative interviews with AMCT patients. We interviewed hospitalized patients who inject drugs to i) explore their perspectives of the NSP and related hospital care; ii) understand factors that may enable or constrain acceptance of supplies; and iii) identify potential strategies to increase uptake.

## Methods

We adopted a focused ethnographic design, which is often applied in research within healthcare settings to answer specific, time-limited research questions in response to a particular problem [37, 38]. In data collection, we used ethnographic interview techniques to probe context and focus on the timely understanding of emic perspectives of participants [39]. Compared to traditional ethnography, focused ethnography can limit or omit participant observation to generate rapid data [37, 38]. A member of our research team (HB) conducted semi-structured interviews with predetermined open-ended questions and probes to rapidly elicit detailed information [39]. The study protocol was developed in consultation with our research team’s community advisory group of people with lived experience of drug use and hospitalization, over half of whom identified as Indigenous; Indigenous Peoples are overrepresented amongst PWID in the study setting.

Eligible participants included admitted inpatients seen by the AMCT who reported recent injection drug use. AMCT staff approached eligible participants and asked about potential participation in the research project. HB approached those interested, obtained written informed consent, and conducted audio-recorded interviews in a location of the participants’ choosing. HB ensured participants’ names and other potentially identifiable information were kept private from AMCT staff. Interviews were conducted between April 20, 2017, and March 7, 2018, and averaged 51 minutes. Participants received $20 CAD for their time and expertise. The audio recordings were professionally transcribed and the transcripts deidentified by HB prior to analysis. We used ATLAS.TI 8 to manage the transcript files and facilitate analysis.

We used latent content analysis to identify and organize the meaning and significance of the transcripts [40]. HB identified clusters of words that related to the same focal meaning and then highlighted, labeled, and sorted those clusters into categories. Data collection and analysis took place iteratively, allowing for initial interpretations and then refinement as interviews continued. This process continued until all transcripts had been analyzed [41]. To ensure rigour, a second member of the research team coded 20% of the transcripts to ensure concordance of interpretation [42]. Additionally, the research team sought feedback from the community advisory group on the validity of the analysis, main findings, and recommendations.

### Ethics statement

This study received human research ethics approval from the University of Alberta’s Health Research Ethics Board (Pro00053613) and operational approval through the Northern Alberta Clinical Trials and Research Centre. All study participants provided written informed consent.

## Results

We completed 25 interviews with patients eligible to access the NSP. Of the 25 participants, 21 accepted supplies prior to their interview. The four participants who did not accept supplies reported that they were not actively injecting in hospital. Participants’ motivations to inject were primarily to manage pain and withdrawal symptoms or due to habit or cravings. For details on the characteristics of the study participants, see Table 1.

**Table 1:** Participant information.

Variable	Descriptive statistics n(%)
**Demographics**	**N = 25**
Gender	
Female	12 (48)
Male	12 (48)
Transgender	1 (4)
Ethnicity	
First Nations, Inuit, or Metis	20 (80)
White	5 (20)
Age	
30–39	7 (28)
40–49	10 (40)
50–59	7 (28)
60+	1 (4)
**Drug use characteristics**	**N = 25**
Length of drug use (years)	
1–10	13 (52)
11–20	4 (16)
21–30	3 (12)
31–40	5 (20)
Type of substance primarily used	
Opioids	16 (64)
Stimulants	4 (16)
Multiple substances	5 (20)
Utilization of drug consumption supplies	
Accepted and used supplies	19 (76)
Did not accept supplies	4 (16)
Accepted but did not use supplies	2 (8)
Injected while hospitalized	
Yes	20 (80)
No	5 (20)
Primary motivations to inject	
Pain or withdrawal symptoms	4 (16)
Habit or cravings	10 (40)
Multiple motivations	5 (20)
Unknown	1 (4)
Not applicable	5 (20)

### Facilitators

#### Motivation to prevent drug-related harm

All participants were familiar with the concept of an NSP and nearly all had utilized one previously in the community. Participants began their hospital stay with awareness of the harms associated with injecting drugs with non-sterile supplies, or they gained knowledge early in their stay from AMCT staff and were thus motivated to use sterile supplies. According to ‘Leah’, “You’re trying to get better when you come to the hospital so having a needle exchange takes away the risk for having another [infection] or making it worse.” Most participants conveyed that they preferred to use sterile supplies while hospitalized. However, several suggested they would use non-sterile supplies if they lost access to the NSP. According to ‘Justin’, “if a person is going to be using dirty needles on the street, what’s going to stop them from using a dirty needle in a hospital?”

#### Trust in the AMCT

Although health was an important motivator, the primary facilitator to accessing the NSP was positive relationships with AMCT staff. Participants described a greater degree of trust in AMCT staff compared to other hospital staff. Participants described AMCT staff as kind and non-judgmental and as promoting collaborative decision-making (e.g., when commencing opioid agonist treatment). Participants particularly appreciated the AMCT’s peer support worker who had lived experience of homelessness and drug use. According to ‘Lydia’, patients trusted the peer support worker because they “ha[d] been down there” and “know where [patients] are coming from.” ‘Allison’ described initially hesitating to accept supplies, but ultimately deciding to accept:

I wanted to get the supplies from [AMCT peer support worker], like the very first time…at the same time I didn’t, because I thought…what’s going on here, right? But, yeah, when he came again I seen that he wasn’t with the [other hospital teams], he was with the [AMCT]. He was with a group of friends. Colleagues.

Positive and trusting relationships with AMCT staff, and especially the peer support worker, appear to have made some participants feel safer accepting supplies.

### Barriers

#### Prior negative experiences in hospital

Despite the trust some participants held in AMCT staff, others feared experiencing negative consequences if they accepted supplies. Participants described negative past experiences and changes to their care when hospital staff had discovered or suspected they used drugs. ‘Curtis’ told us how in the past when he presented to hospital for treatment, the demeanor of hospital staff “changed the minute they found out I was homeless and was a drug addict. They went from being nice and polite to right rude.” Most participants understood that their acceptance of supplies would be communicated to non-AMCT staff and thus anticipated the initiation or exacerbation of negative care due to their participation.

#### Anticipating discriminatory treatment from hospital staff

Specifically, participants anticipated experiencing targeted surveillance of their movements, bodies, or belongings; stigmatizing comments or body language; and involuntary discharge. ‘Owen’ described to us why he initially hesitated to accept supplies:

It’s like, so when [they] asked if I wanted some [supplies], I thought it was some kind of a trick to see if I had anything on me. You don’t know right, told the doctors I’m shooting up and [non-AMCT staff] catch me and kick me out. So, it was kind of weird. It was like, didn’t know whether to trust.

After accepting supplies, several participants attempted to hide their supplies and drug use from non-AMCT staff and described feeling “on pins and needles” or like they were “walking on eggshells” due to anticipation of discovery by non-AMCT staff.

#### Concerns about changes to medication

Participants also feared that accepting NSP supplies would signal that they were continuing to use drugs and result in the non-AMCT attending physician or nursing staff modifying the amount or type of their pain medication provided. Participants experiencing pain feared this would result in a loss of pain control and worsening of their pain while hospitalized.

‘Carl’: If [other staff] find out that a person’s using [the NSP] while they’re getting medications, well then, they’re going to withhold the medications, they’re going to do things different with the medications. I’ve talked to people here in the hospital. They’re not going to tell the doctor that they’re still using because when they get their pill…maybe they’ll change the medication.”

This fear that ‘Carl’ and others described largely resulted from changes to their medication regimes they had experienced in prior hospitalizations, which they perceived were a result of stigma and providers suspecting ongoing unsanctioned drug use.

A small number of participants shared that rather than swallowing their medication, they would instead conceal it in their cheeks. Participants diverted (i.e., “cheeked”) medication to sell, trade, or to inject. These participants were reluctant to accept NSP supplies from AMCT staff because they worried that it would trigger increased surveillance of medication dosing, crushing of medication into food, or switching them to liquid formulations. ‘Allison’ explained:

If I was in [a hospital washroom] and got caught sticking a needle in my arm and what would happen is they would probably change my meds. Or they would put it, mix it in apple sauce…Then I wouldn’t be able to cheek them and use.

Most participants described feeling “degraded”, “singled out”, or “ridiculed” when staff had taken these actions previously. Additionally, the inability to divert medication could produce a need to seek drugs or finances elsewhere, which was described as “not easy” and “frustrating” and could result in participants leaving hospital.

#### Impacts

Most of the 21 participants who accepted supplies reported positive impacts of participating in the NSP. However, a few did report experiencing negative repercussions.

#### Safer drug use

Many participants described the delivery of supplies to their patient rooms as convenient. ‘Rhonda’ struggled with mobility and appreciated how she didn’t have to “worry” about how she was going to access supplies. Participants also described receiving safer drug-use information while receiving their NSP supplies. ‘Justin’ told us:

[The NSP] is a good idea, I think having that kind of visibility for an addict to see that they can approach somebody and ask for supplies or just even talk to them about something like fentanyl. And the [AMCT] has more answers than questions…and a person wouldn’t have to go out and risk their life and trying some kind of a drug or, you know, using a dirty needle.

Nearly all participants described the NSP as increasing their knowledge of and facilitating their ability to participate in safer drug use practices while hospitalized, which was not always easy given their acute illnesses and the hesitation many participants felt in discussing drug use with non-AMCT staff.

#### Engagement in hospital care

Some participants also reported feeling more comfortable completing their treatment because of the NSP.

‘Stephen’: Usually I’d be gone already trying to find myself what I need to get for pain or else just, like, for my addiction. I’d be out there, like, fucking off from the hospital, going AWOL, and getting infected again, and you know, just putting myself at risk, right? And I find it’s good, less easy for me to put myself at risk having the [NSP] here than having it not be here, you know?

A few participants reported that they sought care from this hospital because of the AMCT and its NSP. According to ‘Brandon’, “the only reason that I came back to the hospital was because of the [AMCT and the NSP]…if there was no [AMCT] and I just had to go through the [non-AMCT] doctors, I would have left the hospital”. The NSP thus appeared to both attract and anchor participants to hospital, which may have helped facilitate their completion of medical treatment.

#### Undesired changes to care

However, a small number of participants did experience undesired negative changes to their care. For example, ‘Ruth’ became quite upset when non-AMCT staff confiscated her NSP supplies; according to Ruth, “[non-AMCT nurses] went through my purse when they shouldn’t have…and they found a [needle and syringe]…and the nurse taken out the needles from my purse without consent”. In another instance, ‘Dean’ was diverting and injecting his medication when non-AMCT staff found his supplies, and according to him, “They made me do my meds in applesauce, so I was buying meds in the hospital… I was a little pissed off…but I’m supposed to keep my mouth shut [or] they’ll probably kick me out”.

In most of these instances, however, AMCT staff attempted to intercede and ensure continued access to medication and supplies. ‘Ruth’ described previous experiences reaching out to the AMCT for help addressing issues she faced with non-AMCT staff:

Another few times [non-AMCT nurses] ridiculed me in front of patients, “Open your mouth, open your mouth again.” Trying to make sure my medication was in my mouth going down my throat…That was so embarrassing. I felt like a little kid. [But] if I have a problem [AMCT staff] can help with voicing out and making sure nobody’s discriminating…I don’t really care if they know that I’m an addict. I’m still a human being…I deserve to be treated accordingly.

Despite concerns expressed by some participants of experiencing negative consequences from non-AMCT staff, no participant reported refusing additional NSP supplies. Participants were generally satisfied with the outcome of efforts by AMCT staff to mediate or explain the perceived negative actions taken by non-AMCT staff.

#### Improvements

All participants were asked how the NSP could be improved. First, many participants suggested AMCT staff increase awareness of the NSP and expand access to non-AMCT patients and hospital visitors. Second, participants emphasized a need to be apprised of the implications and consequences of accepting supplies if non-AMCT staff (who were not explicitly required to support access to the NSP) found supplies or drugs among their belongings or caught them injecting or diverting their medication. According to ‘Dean’, patients will accept supplies from AMCT staff when they know “they’re safe getting them.”

Finally, several participants described frustration that they were not supposed to use drugs on hospital property. ‘Ruth’ asked during her interview, “I’m not sure where I’m supposed to go and use in the hospital…Where do we go to use?”. When participants did consume, they often described injecting alone and under stressful, rushed, or otherwise unsafe and non-sterile conditions, including in hospital beds and washrooms, or nearby alleys, trees, or bushes, and at other neighbouring establishments. ‘Oliver’ felt the hospital should have “a safe place to go and do” drugs within the hospital to:

Prevent people from overdosing. Prevent people from getting diseases. A lot of people just end up overdosing or whatever in the bathrooms. People are getting into trouble. Getting arrested…Still not doing it properly. Still sharing needles. I think it would benefit a lot of people…Especially if they’re giving the supplies, they might as well supply a safe place.

Several participants thus implied or stated a need for a dedicated place within the hospital to consume drugs where they could utilize the sterile supplies provided by AMCT staff and yet feel safe from potential negative sanctions from non-AMCT staff.

## Discussion

Empirical research conducted in North America indicates hospital settings may increase risks of harm for PWID, who often continue to use drugs and may encounter stigma, experience poor pain and withdrawal management, and are at increased risk of negative outcomes [16, 17, 20, 26, 43]. Recent scholarship proposes the implementation of hospital policies and interventions that accommodate active drug use, as opposed to explicit or implicit prohibition, to improve outcomes for hospitalized PWID [12, 44–46]. Results from this study support this recommendation. Participants who accessed NSP supplies described the NSP as a trusted source of safer drug-use education and related support, reported a more consistent use of sterile supplies, and expressed greater ease receiving treatment and remaining hospitalized.

However, social forces commonly found within acute care hospitals in North America (e.g., stigma) [47] and risk management policies that influence a healthcare provider’s decision to acknowledge or accommodate active drug use [48] may inhibit or constrain patient-centred harm reduction interventions such as an NSP. These forces were evident in this study. Despite a health authority-wide policy endorsing a harm reduction approach in the care of patients who use substances [49], anticipated stigma or punishment from staff impacted participants’ willingness to access the NSP and accept supplies.

The primary factor that impacted participants’ decisions to access the NSP was trust in AMCT staff. This suggests that all staff should make a concerted effort to build trust with patients by integrating trauma-informed and culturally-safe practices into patient care [50, 51]. One practice hospital staff should adopt is to trust patients’ perceptions of pain and withdrawal symptoms, and ensure immediate access to effective medical management [21, 24]. Hospital administrators should also provide guidance on how staff can recognize the social forces that produce stigma and how they can modify their actions when interacting with PWID (e.g., use non-stigmatizing methods to screen and monitor for drug use) [52, 53]. Hiring staff with lived experience of drug use would facilitate this process [54].

Further modifications to hospital policy may be necessary to facilitate NSP uptake. After accepting supplies, participants described feeling uneasy because they were uncertain of the subsequent ramifications of accepting supplies. To quell patients’ apprehension, hospital administrators may need to establish consistent hospital-wide policies delineating the rights, roles and responsibilities for patients who accept NSP supplies, and of staff caring for those patients. This should include a clear description of any rules or polices governing where (e.g., on or off hospital property) and when (e.g., how soon prior or post medication dispensation) patients should inject if they continue to do so. Hospital policies should also consider providing guidance on medication administration designed to minimize the risks of medications being taken in a non-prescribed way (e.g., the use of liquid oral formulations as is the current practice of the AMCT). Jurisdictions in Canada currently vary in their approach to prescribing oral opioids intended for injection use; however, this type of prescribing was not employed by the AMCT or used in this jurisdiction during the study. Staff may also consider providing instructions regarding how patients may store their drugs [55]. Staff should regularly follow up with patients to ensure they understand the policies and that they are satisfied with their hospital experience.

Additionally, hospitals may choose to establish an inpatient supervised consumption service [12, 56, 57], which several participants described as potentially beneficial. This would address the inconsistencies apparent in the NSP model described within this study where patients were provided with sterile supplies but told they were not allowed to use drugs on site. Supervised consumption services provide a safe and welcoming space where people can use drugs with sterile supplies under the supervision of trained staff without fear of legal prosecution [58, 59]. In part due to the challenges described by participants within this study, the AMCT established a supervised consumption service within the hospital in 2018 [56]. Patients who accept NSP supplies are now encouraged to attend the SCS to consume drugs; drug consumption is still not permitted anywhere else on hospital property. A recent evaluation conducted by some of the authors of this paper found that supervised consumption service participants reported benefits and perceived the space as safer than where they normally would use in hospital [60]. They nevertheless also reported concerns regarding perceived negative social forces within the broader hospital environment and potential impacts on their stay (e.g., changes to their medications). Some refrained from utilizing the service due to these concerns [60]. Further strategies are needed to change hospital culture and support PWID.

Integrating harm reduction interventions such as NSP or supervised consumption services into hospitals where wider social and political forces such as stigma and drug criminalization still exist may not sufficiently reduce harms for hospitalized PWID. Continual efforts must be made to counteract stigmatizing behaviours amongst staff, promote trust and improved communication pathways, and provide patients access to social support and harm reduction services both in-hospital and post-discharge. However, broader societal changes, such as drug decriminalization, may ultimately be necessary.

### Strength and limitations

This study builds on existing literature and fills knowledge gaps. However, participants’ perspectives may be inextricably tied to the AMCT; inpatients who are given access to an NSP without support from a similar specialized team may have different experiences. Additionally, the hospital in which this study was conducted in a large and busy urban hospital in Western Canada and as such, this study may not be reflective of the experiences of hospitalized PWID in substantially different settings.

## Conclusions

In this study, we explored the integration of an NSP into a large, tertiary acute care hospital. Patients described access to sterile injection supplies while hospitalized as beneficial but described occasional negative experiences. Hospitals aiming to improve care for PWID should consider developing hospital policies and interventions that acknowledge the reality of ongoing drug use for some patients and take steps to reduce associated harms. More research is needed on ways to modify the hospital environment to facilitate NSP access and to identify additional acute care interventions that can reduce the health and social inequities experienced by PWID.
